# Comparability of three output prediction models for a compact passively double‐scattered proton therapy system

**DOI:** 10.1002/acm2.12079

**Published:** 2017-04-19

**Authors:** Sven Ferguson, Yong Chen, Clara Ferreira, Mohammad Islam, Vance P Keeling, Andy Lau, Salahuddin Ahmad, Hosang Jin

**Affiliations:** ^1^ Department of Radiation Oncology University of Oklahoma Health Sciences Center Oklahoma City OK USA; ^2^ Department of Radiation Oncology University of Minnesota Medical School Minneapolis MN USA; ^3^ Department of Radiation Oncology Baylor Scott & White Temple TX USA; ^4^ Department of Radiation Oncology CARTI Inc. Little Rock AR USA; ^5^ Oklahoma Cancer Specialists and Research Institute Tulsa OK USA

**Keywords:** double scattering, output model, proton therapy

## Abstract

The purpose of this study was to investigate comparability of three output prediction models for a compact double‐scattered proton therapy system. Two published output prediction models are commissioned for our Mevion S250 proton therapy system. Model A is a correction‐based model (Sahoo et al., Med Phys, 2008;35(11):5088–5097) and model B is an analytical model which employs a function of *r *= (*R’*‐*M’*)/*M’* (Kooy et al., Phys Med Biol, 2005;50:5487–5456) where *R’* is defined as depth of distal 100% dose with straggling and *M’* is the width between distal 100% dose and proximal 100% dose with straggling instead of the theoretical definition due to more accurate output prediction. The *r* is converted to ((*R‐*0.31)‐0.81 × *M*)/(0.81 × *M*) with the vendor definition of *R* (distal 90% dose) and *M* (distal 90% dose‐to‐proximal 95% dose), where *R’* = *R*‐0.31 (g cm^−2^) and *M’* = 0.81 × *M *(g cm^−2^). In addition, a quartic polynomial fit model (model C) mathematically converted from model B is studied. The outputs of 272 sets of *R* and *M* covering the 24 double scattering options are measured. Each model's predicted output is compared to the measured output. For the total dataset, the percent difference between predicted (P) and measured (M) outputs ((P‐M)/M × 100%) were within ±3% using the three different models. The average differences (±standard deviation) were −0.13 ± 0.94%, −0.13 ± 1.20%, and −0.22 ± 1.11% for models A, B, and C, respectively. The *p*‐values of the t‐test were 0.912 (model A vs. B), 0.061 (model A vs. C), and 0.136 (model B vs. C). For all the options, all three models have clinically acceptable predictions. The differences between models A, B, and C are statistically insignificant; however, model A generally has the potential to more accurately predict the output if a larger dataset for commissioning is used. It is concluded that the models can be comparably used for the compact proton therapy system.

## 1 Introduction

Current treatment planning systems (TPS) do not provide accurate monitor units (MUs) used to deliver the prescribed dose for passively scattered proton therapy systems.^1^ Therefore, output prediction models are needed to convert the prescribed dose to MU. This is mainly due to the variety of models and vendors of proton therapy systems currently available. Additionally, different systems have varying proton acceleration and extraction designs, beamline designs used to shape the beam, and nozzle delivery techniques. Combinations of these variations add uncertainty in output prediction models across different systems.

Various approaches of output (cGy/MU) models have been employed for proton therapy systems. A correction‐based model such as the model proposed by Sahoo et al.^2^ utilizes the multiplication of correction factors based on beam parameters (option, range, and modulation width) that can independently change the output in the model. In contrast, analytical models^3^, ^4^, ^5^, ^6^ use mathematically derived analytical formulas preferably combined with empirical fitting parameters that are fitted to the measured data. In addition, Hotta et al.^7^ used a simplified Monte Carlo simulation to calculate MU for a passively scattered proton therapy system. In the model, the output is predicted by a product of the output measured in a standard condition and a clinical beam delivery condition factor (F_calc,clinical_). The F_calc,clinical_ is a multiplication of three factors: a beam‐spreading device factor (F_BSD_), a patient‐specific device factor (F_PSD_), and a field size correction factor (F_FS_). The F_BSD_ is measured in the phantom, while F_PSD_ and F_FS_ are calculated using the simplified Monte Carlo simulation.

Recently, we commissioned the correction‐based model^2^ and one of the analytical models^4^ for our compact double‐scattered S250 proton therapy system (Mevion Medical Systems, Littleton, MA, USA). In addition, a new model^8^ mathematically derived from the analytical model was commissioned. The purpose of this research is to investigate the comparability of the three output prediction models for the compact proton therapy system.

## 2 Methods

### 2.A Proton therapy system

The S250 proton therapy system is a single‐room proton therapy system consisting of a synchrocyclotron which accelerates proton beams at a nominal energy of 250 MeV on a rotating gantry, a double scattering field shaping system (FSS), and a six‐degree‐of‐freedom (6‐DOF) robotic patient couch system. The beam delivery system has 24 total options with clinical ranges (*R*) from 5.0 to 32.0 g cm^−2^. The *R* is defined as depth of the 90% dose point on the distal falloff in a normalized percent depth dose (PDD) scan and the modulation width (*M)* is defined as the width between the distal 90% and proximal 95% dose points of the PDD curve.

These options are separated into three general field types. The first option group is denoted “large” (options 1 to 12) with *R* from 5.0 to 25.0 g cm^−2^ mainly delivered by a large applicator with a maximum field size of 25 cm diameter. This option group uses six modulation wheels where every two options share the same modulation wheel, five high‐Z first scatterers, and one second scatterer. The ranges are shifted using two sets of final absorber plates after the second scatterer (coarse in steps of 0.5 g cm^−2^ and fine in steps of 0.1 g cm^−2^) whose step resolution is 0.1 g cm^−2^ with a maximum change in range within an option 2.4 g cm^−2^. The *M* for the large option group ranges from 2.0 to 20.0 g cm^−2^ in steps of 0.1 g cm^−2^.

The second option group is denoted “deep” (options 13–17) with *R* from 20.1 to 32.0 g cm^−2^ delivered by a small applicator of the maximum field size of 14 cm diameter. This group shares one modulation wheel and a unique combination of one first scatterer and one second scatterer. The ranges are shifted using a static absorber wheel, with varying thicknesses by means of a circular wedge downstream of the modulation wheel set in the beam line for each option, and the final absorber plates. The *M* for the deep group ranges from 2.0 to 10.0 g cm^−2^ in steps of 0.1 g cm^−2^.

The third group is denoted “small” (options 18 to 24) with *R* from 5.0 to 20.0 g cm^−2^ delivered by the small applicator. The *M* for the small group ranges from 2.0 to 20.0 g cm^−2^ in steps of 0.1 g cm^−2^. Each small option has a unique combination of a modulation wheel (total 7 wheels), one first scatterer (0.0 g cm^−2^ thickness for our system), and one second scatterer. The ranges are shifted using a static absorber wheel upstream of the modulation wheels rotated to a particular thickness corresponding to a range. Note that the combination of modulation wheels and scatterers for options is machine specific.

### 2.B Correction‐based model

A correction‐based model was proposed by Sahoo et al.^2^ and modified to predict the output for our Mevion system as follows:

(1) $$\Psi_{\text{A}}=\Psi_{\text{o}}\cdot \mathrm{ROF}\cdot \mathrm{SOBPF}\cdot \mathrm{RSF}\cdot \mathrm{SOBPOCF}\cdot \mathrm{OCR}\cdot \mathrm{FSF}\cdot \mathrm{ISF}\cdot \mathrm{GACF},$$

where $\Psi_{A}$ (originally defined as (d/MU)_wnc_; $\Psi_{A}$ is used instead for comparison with the other models) is the output (cGy/MU) in water without range compensator, $\Psi_{o}$ is the absolute output for a reference field (this is added to the original model since the output for our reference field for option 20 with *R* = 15.0 g cm^−2^ and *M* = 10.0 g cm^−2^ is 1.06 cGy/MU), ROF is the relative output factor normalized to the reference field, SOBPF is the spread‐out Bragg peak (SOBP) factor, RSF is the range shifter factor, SOBPOCF is the SOBP off‐center factor, OCR is the off‐center ratio, FSF is the field size factor, ISF is the inverse square factor, and GACF is the gantry angle correction factor that is unique to the Mevion S250 system. The correction‐based model employs a large dataset obtained by fixing the beam parameters at reference conditions then incrementally changing one or more parameters while measuring the output at each increment. The output is then converted into a factor by taking the ratio of the measured output to the output of the reference conditions. The factors are then tabulated and either 1‐D or 2‐D linear interpolation is performed for data points that are not directly measured.

The output for commissioning the models, as well as validation, is measured with a Farmer‐type chamber (TN30013, PTW, Freiburg, Germany) in a water tank (Blue Phantom, IBA, Louvain‐La‐Neuve, Belgium). A constant air gap of 10 cm is used for all measurements without compensator. The large options use the large applicator with an open field size of 20 × 20 cm^2^ and the deep and small options use the small applicator with an open field size of 10 × 10 cm^2^.

#### 2.B.1 Relative output factor (ROF)

The ROF is an option‐specific parameter that accounts for relative changes in output when any option other than the calibration option (option 20 with *R* = 15.0 g cm^−2^, *M* = 10.0 g cm^−2^) is used. It is the ratio of the measured output of a selected set of *R* and *M* for an option to that of the calibration option. Changes in the output for options are largely due to different beamline configurations. In this study, the deepest *R* for each option is used with *M* of 10.0 g cm^−2^ for ranges larger than 10.0 g cm^−2^ or a modulation width slightly less than the range for ranges less than 10.0 g cm^−2^ (a total of 24 measurements; see Table 1).

**Table 1:** Relative output factors (ROFs) for all options. Additionally, the range (largest range in the option), modulation, and applicator field sizes are shown that were used for measurements

Option	Range (g cm^−2^)	Modulation (g cm^−2^)	Field size (cm^2^)	ROF
1	25.0	10.00	20 × 20	1.22
2	22.5	10.00	20 × 20	1.17
3	20.8	10.00	20 × 20	1.13
4	18.7	10.00	20 × 20	1.08
5	16.7	10.00	20 × 20	1.02
6	14.8	10.00	20 × 20	0.97
7	13.1	10.00	20 × 20	0.89
8	11.4	10.00	20 × 20	0.82
9	9.9	8.00	20 × 20	0.83
10	8.5	6.00	20 × 20	0.87
11	7.2	6.00	20 × 20	0.79
12	6.0	4.00	20 × 20	0.86
13	32.0	10.00	10 × 10	1.35
14	29.5	10.00	10 × 10	1.32
15	27.0	10.00	10 × 10	1.29
16	24.5	10.00	10 × 10	1.26
17	22.0	10.00	10 × 10	1.21
18	20.0	10.00	10 × 10	1.12
19	17.7	10.00	10 × 10	1.06
20	15.0	10.00	10 × 10	1.00
21	13.2	10.00	10 × 10	0.94
22	11.1	10.00	10 × 10	0.94
23	9.0	8.00	10 × 10	0.95
24	6.9	6.00	10 × 10	0.96

#### 2.B.2 Spread‐out Bragg peak factor (SOBPF)

The SOBPF accounts for changes in output for modulation widths that differ from the ROF modulation width within an option. It is measured for each option by fixing the *R* (the largest *R* within the option) and varying the *M* from 2 g cm^−2^ to the maximum *M* for the option in steps of 2 g cm^−2^. The SOBPF is then found by taking the ratio of the output for the desired *M* to that of the reference ROF modulation width within the option. Due to relatively large output change between 2 and 4 g cm^−2^ modulation widths a SOBPF for 3 g cm^−2^ is also obtained (a total of 182 measurements).

#### 2.B.3 Range shifter factor (RSF)

The RSF accounts for output changes within an option due to range shift. The thicknesses of the final absorber plates and one of the absorber wheels are dictated by the selected *R* and *M*. For this reason, the RSF is a function of *R* as well as *M*. The RSF is determined by measuring the output of three ranges per option (the minimum, median, and maximum *R*) with varying *M*s per range (a total of 324 measurements with minimum, maximum, and various intermediate modulation widths; see Table 2). The RSF is determined for an option by 2‐D linear interpolation of *R* and *M* in Matlab (Mathworks, Natick, MA; version R2013b). For some options, the maximum *M* is greater than for the minimum *R*. For instance, the minimum *R* for option 12 is 5.0 g cm^−2^; however, the maximum *M* is 6.0 g cm^−2^. If the modulation exceeded the range, it would extend outside of the patient (e.g., for *R* = 10.0 g cm^−2^ and *M* = 11.0 g cm^−2^, a high dose region of 1.0 g cm^−2^ will be outside of the patient). For this reason an R < M is not an allowable parameter entry into the Mevion system. Because of this the output cannot be measured for this set, but for 2‐D interpolation in Matlab it is required to have the RSF for the set. In this case, the value is extrapolated by a log fit using the outputs measured with the same minimum *R* and other intermediate modulation widths.

**Table 2:** Range shifter factor (RSF) for options 1, 13, and 20

Modulation (g cm^−2^)						
Option	Range (g cm^−2^)	2.0	10.0	20.0		
1	25.0	1.0000	1.0000	1.0000		
	23.8	0.9914	0.9931	0.9895		
	22.6	0.9823	0.9855	0.9772		
13	Range (g cm^−2^)	2.0	5.0	10.0		
	32.0	1.0000	1.0000	1.0000		
	30.8	1.0011	1.0010	1.0001		
	29.6	1.0018	1.0014	0.9994		
20	Range (g cm^−2^)	2.0	10.0	13.3	14.3	15.3
	15.3	1.0000	1.0000	1.0000	1.0000	1.0000
	14.3	0.9729	0.9680	0.9589	0.9535	0.9665^a^
	13.3	0.9450	0.9336	0.9116	0.9196^a^	0.9251^a^

a The placeholder values for option 20 are extrapolated for use only in 2‐D interpolation and are not used in the model.

#### 2.B.4 SOBPOCF and ISF

**Figure 1:**
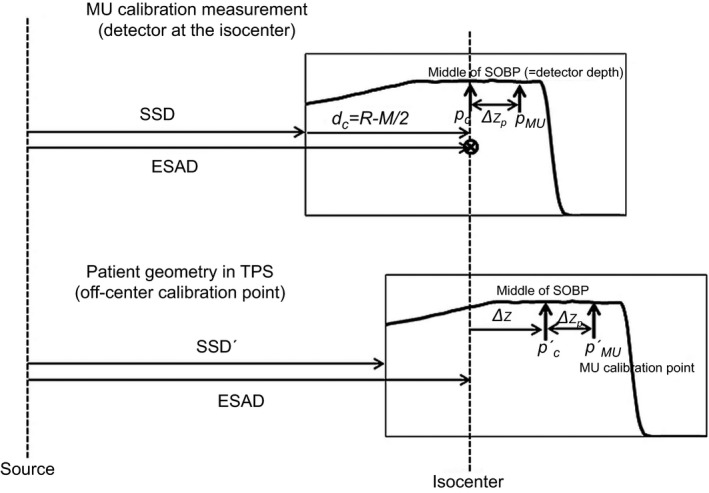
Geometry conversion for ISF_OCR_ from calibration geometry (isocenter = depth of middle of the SOBP = detector) to patient geometry (off‐center and off‐isocenter).

The SOBPOCF takes into account the relative changes in output when the measurement point is located longitudinally (along the beam line) away from the center of the SOBP. The SOBFOCF for a fixed source to detector distance (SDD) is given:

(2) $$SOBPOCF=PDD\frac{{\left(SSD+d_{p}\right)}^{2}}{{\left(SSD+d_{c}\right)}^{2}},$$

where *PDD* is the percent depth dose normalized at the center of the SOBP, *SSD* is the source to surface distance used for the *PDD* measurement, *d*_*p*_ is the depth of MU calibration, and *d*_*c*_ is the depth of center of the SOBP. This factor is the dose at the calibration point relative to center of the SOBP with a fixed *SDD* (detector at the isocenter for both measurements in the published model). The ISF takes into account an MU calibration point that is located away from the isocenter:

(3) $$ISF={\left(\frac{SAD}{SPD}\right)}^{2},$$

where *SAD* is the source to axis (isocenter) distance and *SPD* is the source to point of MU calibration distance. We propose combining SOBPOCF and ISF in this study for several reasons. First, the PDD is cumbersome to measure for all different sets of *R* and *M* with a fixed SSD. Second, the *SAD* for our system is option‐specific and thus different effective source to axis distance (*ESAD*) should be used for each option. Third, to minimize the setup error in our institution, the MU calibration is not performed with a fixed SDD with off‐center distance, but always at the isocenter and center of the SOBP (i.e., *SDD *= *ESAD* and chamber depth = *R*‐*M*/2) and corrected for off‐center and off‐isocenter changes. In theory, the outputs at *p*_*c*_ (point at center of the SOBP) and *p*_*MU*_ (off‐center point) are the same in the isocentric setup (isocenter at the depth of *R*‐*M*/2) when the *p*_*MU*_ is on the flat SOBP [*Ψ*(at *p*_*c*_)=*Ψ*(at *p*_*MU*_)] as shown in Fig. 1. The output at *p’*_*MU*_ (MU calibration point in patient plan from the TPS) is inversely proportional to the distance squared from the source between *p*_*MU*_ and *p’*_*MU*_. The output at *p’*_*MU*_ is given by:

(4) $$\Psi \left(atp\prime_{MU}\right)=\Psi \left(atp_{MU}\right)\frac{{\left(ESAD-\Delta z_{p}\right)}^{2}}{{\left(ESAD-\Delta z_{p}-\Delta z\right)}^{2}}=\Psi \left(atp_{c}\right)\frac{{\left(ESAD-\Delta z_{p}\right)}^{2}}{{\left(ESAD-\Delta z_{p}-\Delta z\right)}^{2}}=\Psi \left(atp_{c}\right)\times ISF_{OCF},$$

where *ISF*_*OCF*_ $[={\left(ESAD-\Delta z_{p}\right)}^{2}/{\left(ESAD-\Delta z_{p}-\Delta z\right)}^{2}]$ is the *ISF* with the SOBP off‐center correction, $\Delta z_{p}$ ((‐) for downstream and (+) for upstream) is the distance (parallel to the beam) of the calibration point *p’*_*MU*_ from the center of the SOBP, and Δz is the offset when the center of the SOBP and the isocenter are not located at the same point. The error due to this modification is limited since the calibration point is usually selected close to the center of the SOBP (a few centimeters compared to nominal SAD = 200 cm) and SOBPOCF is very close to unity for the most of cases as shown in the previous publication.^2^

#### 2.B.5 Off‐center ratio (OCR)

The OCR allows for lateral shifts of the measurement points on a plane perpendicular to the beam away from the central axis. A lateral shift can be applied in order to avoid the fluence and scatter perturbation from the block edge regions and allow measurements within the open field. The OCR for each option is measured with a PTW Octavius 729 XDR 2‐D ion‐chamber array located at the isocenter and center of the SOBP for each option with the same *R* and *M* for ROF (a total of 24 measurements; Table 1). The profile data are then tabulated to be used for 2‐D linear interpolation once the distances (x, y) from the central axis are found in a beam's eye view from the TPS.

#### 2.B.6 Field size factor (FSF)

The field size factor accounts for relative scatter changes due to changing the collimated open field size. Our proton system does not have rectangular variable collimators, and thus the FSF entirely relies on the brass block cutout. It is found by taking the ratio of the output of a field size to the reference field size (10 × 10 cm^2^ for small and deep option groups and 20 × 20 cm^2^ for large option group). The outputs for three options with the deepest *R* and intermediate *M* within each option group are measured (a total of 54 measurements). The field sizes projected on the isocenter plane are approximately 3.5 cm diameter (circular), 5 × 5 cm^2^, 10 × 10 cm^2^, 14 cm diameter (circular), 20 × 20 cm^2^ (large option only), and 25 cm diameter (circular; large option only).

#### 2.B.7 Gantry angle correction factor (GACF)

The Mevion S250 system is unique in its design by the fact that the entire cyclotron rotates with the external gantry. This causes a variation in output depending on the gantry angle (total rotation of 190° from 355° to 185° with 0° facing down).^9^ The gantry angle correction factor (GACF) is thus introduced to account for this variation by calculating the ratio of the output to the calibration beam angle (gantry angle = 0^o^, facing downwards) to that of the clinical gantry angle:

(5) $$GACF=\frac{d/MU_{\left(clinicalgantryangle\right)}}{d/MU_{\left(gantryangle=0^{\circ }\right)}}$$

This is obtained using the Farmer‐type chamber placed at the isocenter inserted in an acrylic compensator phantom provided by the vendor. Due to the limit of the phantom size (buildup to the chamber is 10 g cm^−2^), the GACF data points are taken for one or two options within each option group for every 15 degree (large: options 3 and 5, deep: option 17, and small: options 18 and 20; a total of 75 measurements).

#### 2.B.8 MU calculation

The output in this study (model A) is thus given:

(6) $$\Psi_{A}=\Psi_{o}\cdot \mathrm{ROF}\cdot \mathrm{SOBPF}\cdot \mathrm{RSF}\cdot {\mathrm{ISF}}_{\mathrm{OCR}}\cdot \mathrm{OCR}\cdot \mathrm{FSF}\cdot \mathrm{GACF}$$

In order to eliminate uncertainties due to fluence perturbation by range compensator and patient scatters, the output is measured without range compensator in the water tank. As demonstrated by Sahoo et al.,^2^ in clinical practice a verification plan using a virtual water phantom without range compensator is generated from a patient plan with the same geometry (a calibration point located at the same water equivalent depth, that includes the water equivalent thickness of the range compensator along the ray between the source and the point). The MU to deliver the prescribed dose at the calibration point in patient plan is given:

(7) $$\begin{array}{cc} MU & =D_{pp}/\Psi_{p}=D_{vpnc}/\Psi_{wnc}\\ & =D_{vpnc}/\left(\Psi_{o}\cdot \mathrm{ROF}\cdot \mathrm{SOBPF}\cdot \mathrm{RSF}\cdot {\mathrm{ISF}}_{\mathrm{OCR}}\cdot \mathrm{OCR}\cdot \mathrm{FSF}\cdot \mathrm{GACF}),\right) \end{array}$$

where D_pp_ is dose at the calibration point in patient plan, *Ψ*_*p*_ is the output at the point in patient, *Ψ*_wnc_ = *Ψ*_A_, and D_vpnc_ is dose in the verification plan without compensator at the calibration point.

### 2.C Analytical models

The second model (model B) is the analytical model^2^ as a function of *r*:

(8) $$\Psi_{B}\left(r\left(R,M\right)\right)=\frac{CF\times \Psi_{o}\times D_{c}}{100/\left(1+a_{0}r^{a_{1}}\right)}\times \left[s_{0}+s_{1}\left(R-R_{L}\right)\right]\times ISF_{OCF}\times OCR\times FSF\times GACF,$$

where *r* is (*R’*‐*M’*)/*M’* (*R’* = range of distal 100% dose and *M’ *= width between distal 100% dose and proximal 100% dose with straggling in the measured *PDD*), *CF* is a correction factor for the relative output changes per option*,* $\Psi_{o}$ is the output factor of the reference field, $D_{c}$ is the entrance dose in the calibration reference geometry, $R_{L}$ is the lowest *R* for the option, and *a*_*o*_ *, a*_*1*_ *, s*_*o*_ *,* and *s*_*1*_ are option‐specific fitting parameters. The *r* is modified to ((*R –* 0.31) *–* 0.81 × *M*)/(0.81 × *M*)) with the Mevion definition of *R* (depth of distal 90% dose) and *M* (width between distal 90% dose and proximal 95% dose), where 0.31 g cm^−2^ is the mean difference between *R* and *R’* (*R’* = *R* – 0.31) and 0.81 is a mean ratio of *M’* and *M* (*M’* = 0.81 × *M*) for a total of 230 SOBP scans in all 24 options. The modification was necessary for more accurate output prediction due to mismatch between nominal (vendor definition), theoretical (without straggling), and actually measured values of *R* and *M*. The derivation can be found in the appendix of our previous publication.^8^

An additional quartic polynomial fit (model C) mathematically converted from the model B (inspired by the Taylor series) is also examined:

(9) $$\Psi_{C}\left(r\left(R,M\right)\right)=\left(p_{0}+p_{1}r+p_{2}r^{2}+p_{3}r^{3}+p_{4}r^{4}\right)\times \left[s_{2}+s_{3}\left(R-R_{L}\right)\right]\times ISF_{OCF}\times OCR\times FSF\times GACF,$$

where *p*_*0*_, *p*_*1*_, *p*_*2*_, *p*_*3*_, *p*_*4*_, *s*_*2*_, and *s*_*3*_ are option‐specific fitting parameters. The correction factors (OCR, FSF, and GACF) are added to the original analytical models to have the equivalent formula.

To fit the basic model (the first term on the right side of models B and C), the same dataset for SOBPF for model A (absolute outputs) is used. The output change by effective source shift due to range change within an option (the second term) is fit by three measurements for each option (maximum, medium, and minimum *R* with corresponding *M* for the same value of *r*). All the option‐specific parameters for models B and C are fit by the Matlab curve fitting toolbox. Implementation of the analytical models in detail can be found in our previous publication.^8^

### 2.D Output measurements for validation

To test the models for accuracy and compare them against each other, the outputs of 272 sets of *R* and *M* covering the 24 options are measured. These are selected from treatment plans (for typical clinically used *R* and *M* combinations) as well as random sets of *R* and *M* (some of which are rarely selected for clinical beams, but are measured to cover a wide array of *R* and *M* combinations) and are measured independently of the model fit data points. Most of the points of measurement for the validation are located at the center of the SOBP and the isocenter. However, some measurement points are away from the center of the SOBP (maximum ± 5 cm offset of the middle of the SOBP) and/or the isocenter (60 measurements). The outputs predicted by the models are compared to the measured validation outputs (the percent difference = (Prediction – Measurement)/Measurement × 100%). Additionally, each model is compared against each other using the Student's t‐test.

## 3 Results

### 3.A Correction factors

**Figure 2:**
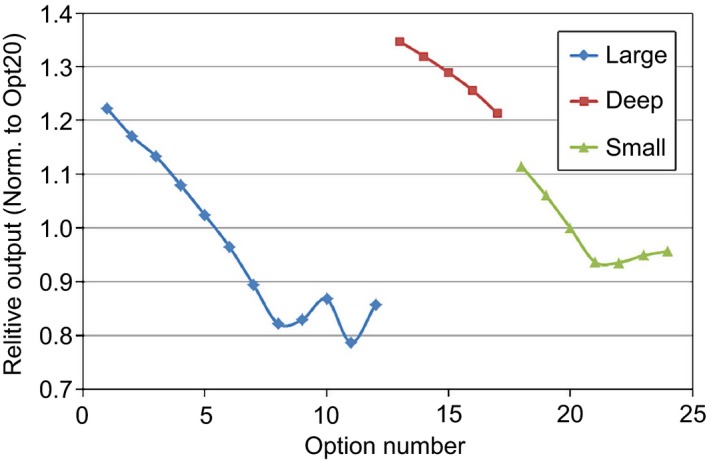
Relative output factors (ROFs) for all options.

Figure 2 shows the ROFs for all options. All options are normalized to the reference option 20. The large option group (options 1–12) has the largest spread of ROFs ranging from 1.22 to 0.86, the deep option group (options 13–17) has the least varying ROFs ranging from 1.35 to 1.21, while the small option group (options 18–24) has ROFs ranging from 1.12 to 0.95.

**Figure 3:**
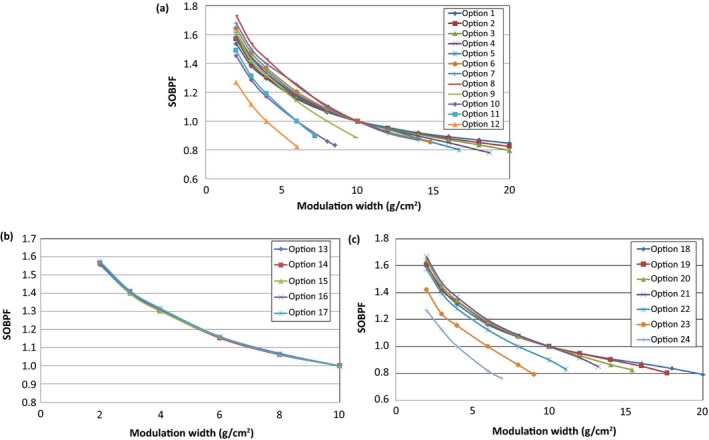
Spread‐out Bragg peak factor (SOBPF) for large (a), deep (b), and small (c) option groups.

Figure 3 displays the SOBPFs for the large (a), deep (b), and small (c) option groups. The large and small option groups show large changes in SOBPF values for small modulations (M < 10 g cm^−2^). For these same option groups, larger modulations (M > 10 g cm^−2^) show little changes in SOBPF. The deep option group shows very little deviation between each option. This is mainly due to sharing the same FSS devices (a first scatterer, a modulation wheel, and a second scatterer) for all options.

Table 2 shows the RSFs selected for options 1 (large option), 13 (deep option), and 20 (small option). The largest *R* for each option is set as the reference *R*, therefore RSF = 1.00. Deep options tend to have RSFs that deviate little from unity (up to 1.6% for option 17). The large option group shows changes in RSF up to 13.6% (option 12) while the small option group shows changes in RSF up to 16.5% (option 24). In general, the smallest *R* within an option group shows the largest RSF change.

**Figure 4:**
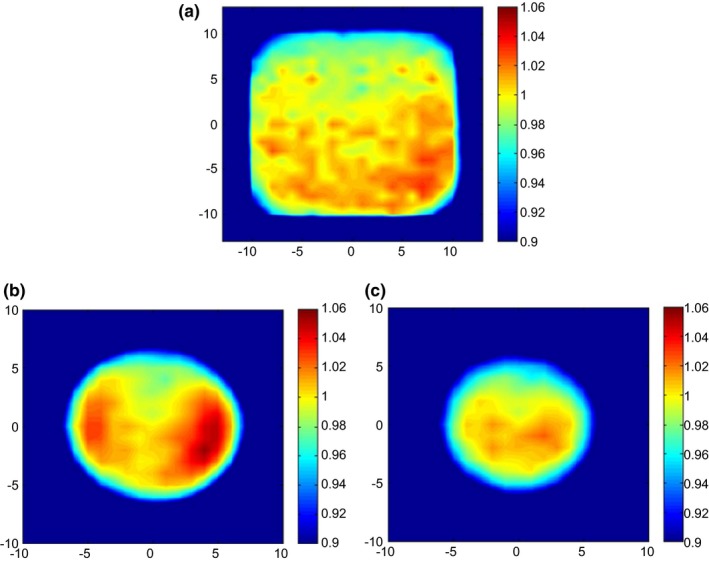
Off center ratio (OCR) for (a) option 1, (b) option 13, and (c) option 18 (field size unit: cm).

Figure 4 show a 2‐D distribution of OCR for option 1 (large), option 13 (deep), and option 18 (small). The 20 × 20 cm^2^ was used for large options, while a maximum circular field available for each option was used for deep and small options. The OCR measurement with the 2‐D ion‐chamber array (the center‐to‐center detector spacing = 10 mm) was resampled at a resolution of 0.5 mm/pixel and tabulated for interpolation. Each point away from the central axis was normalized to the central axis measurement (OCR at central axis = 1.00). The OCRs were not radially symmetric as shown in Fig. 4, and thus the 2‐D interpolation is clinically used for off‐axis calibration points.

The field size factors (FSFs) of various field sizes for selected options are shown in Table 3. It can be seen that field sizes greater than 5 × 5 cm^2^ have an FSF that is near unity for most of the options, while field sizes less than or equal to 5 × 5 cm^2^ have large variations. For shallow depth with the small field sizes the outputs are higher than expected. This is due to the slit scattering effect which originates from protons scattered from aperture edges. These low‐energy scattered protons contribute to a dose bump along the field edges where the outputs increase with the small field sizes at the central axis for the shallow options (options 12 and 24). In practice, to alleviate the uncertainty by the field size in the MU calculation, it is highly recommended to use patient‐specific apertures when D_vpnc_ is calculated in the verification plan.

**Table 3:** Field size factor (FSF) for selected options

Option	Field size					
	3.5 cm diameter circular	5 × 5 cm^2^	10 × 10 cm^2^	14 cm diameter circular	20 × 20 cm^2^	25 cm diameter circular
1	0.687	0.985	1.000	1.000	**1.000**	1.000
6	0.868	1.006	1.004	1.000	**1.000**	0.996
12	0.903^a^	1.019^a^	1.016	0.999	**1.000**	0.999
13	0.656	0.982	**1.000**	1.000	1.000	1.000
15	0.700	0.971	**1.000**	1.000	1.000	1.000
17	0.778	0.984	**1.000**	1.000	1.000	1.000
18	0.758	0.989	**1.000**	1.000	1.000	1.000
20	0.938	1.003	**1.000**	1.000	1.000	1.000
24	1.015^a^	1.011^a^	**1.000**	1.000	1.000	1.000

a Relatively higher readings may come from the slit scattering for the detector at shallow depths. The reference field sizes are marked in bold.

**Figure 5:**
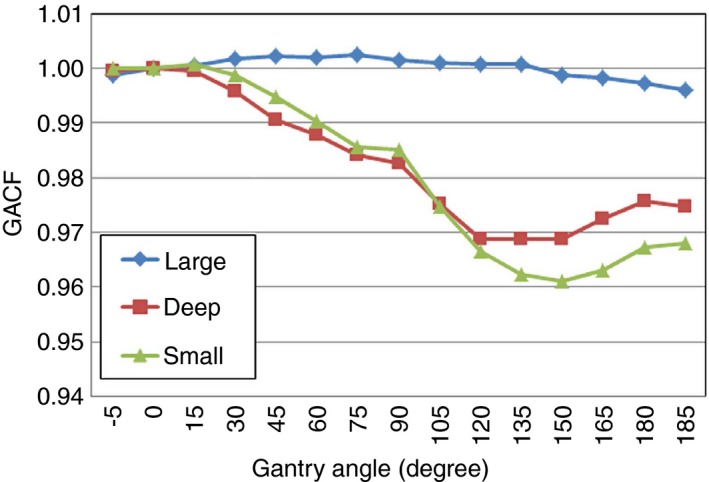
Gantry angle correction factors (GACFs) for all large, deep, and small option groups.

The GACF varies little among all options within an option group; therefore, all options within a group share the same factor (i.e., three different factors for each gantry angle are used). The large options have very little gantry angle dependence and hardly deviate from unity, as shown in Fig. 5. The output for deep options vary up to 2.3% (maximum deviation at angle 135°) from the calibration condition (gantry angle = 0°) while the output for small options varies up to 3.6% (maximum deviation at angle 150°) from calibrations conditions.

### 3.B Validation measurements and model comparison

**Figure 6:**
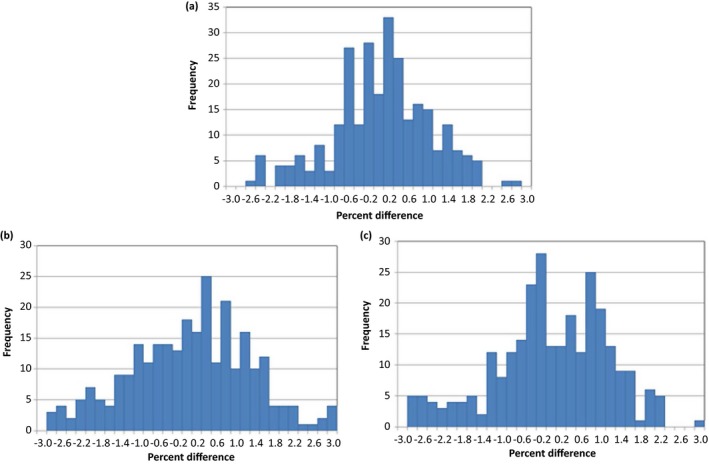
Percent difference between predicted and measured outputs for (a) model A, (b) model B, and (c) Model C.

For the total 272 dataset, the percent differences fell within ± 3% for the three different models. Figure 6 shows histograms of the percent differences. The average differences (± SD) were −0.13 ± 0.94%, −0.13 ± 1.20%, and −0.22 ± 1.11% for models A, B, and C, respectively. The *p*‐values of the t‐test were 0.912 (model A vs. B), 0.061 (model A vs. C), and 0.136 (model B vs. C), showing statistically insignificant differences between any of the three models.

## 4 Discussion

The correction‐based models, such as model A, tend to have better output predictions (smaller SD) than analytical models. This is due to the fact that the correction‐based models use the measured data directly through linear interpolation. In contrast, the fitting parameters in the analytical models do not fit the measured data perfectly at all points. Therefore, the analytical models may have varying accuracies across options even when using the same dataset as a correction‐based model. In addition, the range shifter slightly changes the output within an option. It is modeled using 2‐D interpolation (RSF) within an option in the correction‐based model; whereas a linear fit (the second term in the models) is used for all sets of *R* and *M* within the option in the analytical models to fit a nonlinear dataset adding about 1.0% uncertainty. A total of 12 points out of 272 measurements (0.4%) had more than 2% errors using the model A. These high deviations were distributed over seven different options. Most of them (nine points) had the range less than 10 g cm^−2^ and half of them (six points) had relatively narrow modulation (< 4 g cm^−2^). When the modulation is small, the SOBP is not entirely flat and thus usually the output measurement has higher statistical deviation. In this situation, more data collection will not significantly improve the accuracy of model fitting and extra care (phantom setup and detector position) should be taken when the output is measured.

Uncertainties can arise from a variety of reasons when commissioning the models. For instance, the placement of the chamber can cause slight output changes in the case of small modulation widths (usually *M* < 4 g cm^−2^) where the modulation may not be flat. For these cases, careful placement of the chamber should be taken in order to minimize the gradient effect^10^ on the data that are used in the output models, which in turn will affect the output predictions. As noted in Fig. 3, there are regions within the SOBPFs that have relatively large changes between measured points. Since linear interpolation is used to determine the SOBPF for modulation widths that are not directly measured, these steep changes between measured points can be sources of uncertainty. In these regions the SOBPF may not be linearly decreasing with an increase in *M*. Should a region between measured points not have acceptable predictions due to modulation changes, one should measure additional SOBPFs for these regions. In this research, this issue occurred between modulation widths of 2 and 4 g cm^−2^. An added measurement for all options at 3 g cm^−2^ was found to provide acceptable output predictions. For instance, a 2.8% difference was calculated between SOBPFs with and without output measurement at 3 g cm^−2^ for option 8. It is estimated that, once the measurements are added into the model, uncertainty is less than 1% due to this issue. Similar to the SOBPF, the RSF is susceptible to uncertainty of linear interpolation. However, the 2‐D interpolation for RSF has the potential to alleviate the issue. For our research, uncertainty associated with the RSF is estimated to be less than 1%.

It was found that if a 1‐D interpolated OCR is used for an off‐axis calibration point assuming a radially symmetric beam, there will be up to 6% dosimetric error for an extreme case (i.e., OCR = 0.97 vs. 1.03 for mirror points toward beam edge) for our system as shown in Fig. 4. The radial asymmetry may stem from the absorber wheels which have varying thicknesses by means of a circular wedge and partial shining of a beam on the modulating wheel steps. It is of great importance to investigate the actual beam symmetry for accurate output prediction when the calibration point is off axis especially for off‐axis small patch beams.

In this study, only one ESAD is employed for each option which ranges from 171.9 cm (option 22) to 180.8 cm (option 13). The most extreme ESAD shift (up to 5 cm within an option due to range shift) contributes only a ± 0.5% uncertainty for ISF_OCF_. For instance, with a nominal *ESAD* = 185 cm and actual *ESAD* = 180 cm with a +5 cm shift of calibration point from the isocenter, the dosimetric output change is a factor of nominal 1.0563 (= [(185 + 5)/185]^2^) versus actual 1.0585 (= [(180 + 5)/180]^2^) which results in a 0.2% difference. Therefore, in most cases, the uncertainty arising from using one ESAD per option is acceptable (< 0.5%).

For small field sizes (≤5 × 5 cm^2^), it is required to directly measure the output due to inaccurate calculation of scatter contributions by pencil beam algorithm used in the TPS and irregular shape of aperture for actual clinical beams. Additionally, it is highly discouraged to use an aperture with radius of <1 cm for deep seated targets since the depth dose curve of a proton beam significantly deviates from the distinct Bragg peak due to the multiple Coulomb‐scattering effect, and thus the surface dose is higher than the target dose.^11^ For large field sizes (> 5 × 5 cm^2^), square‐field apertures (10 × 10 cm^2^ for the small applicator and 20 × 20 cm^2^ for the large applicator) can be used for clinical beams with uncertainty of up to 1% for most of the beams as shown in Table 3.

The Mevion S250 cyclotron is mounted on an external gantry located behind a wall adjacent to the treatment room. During beam preparation the external gantry rotates to match the positioning angle of the inner gantry (applicator). The gravitational sag due to the various cyclotron positions results in slight changes in the energy. These energy shifts are accompanied by SAD shifts and beam shape changes. An energy modulation disk (EMD) inside the cyclotron system is used to correct for these changes as well as to steer the beam. It is believed that the subtle correction of the beam by the EMD, depending on the gantry angle, results in the variations of output. It is for these reasons that changing gantry angles can cause a maximum output change of 3.6%. Because one GACF (an average of several options within a group) was used for each option group, an error up to 0.5% was found. It should be noted that these changes in output are system specific.

The outputs are measured in the water phantom without patient‐specific range compensators. There is intrinsic uncertainty associated with conversion of absorbed dose in a water phantom without compensator for calibration measurement to absorbed dose in a patient. It can be accounted for using compensator and patient scatter factors (CSF and PSF; CPSF = CSF × PSF) as shown in Sahoo et al.'s publication.^2^ For MU calculation, these are cancelled out by calculating dose in a virtual water phantom without range compensator in TPS (i.e., D_pp_ = D_vpnc_·CSF·PSF and Ψ_*p*_ = Ψ_wnc_·CSF·PSF in Eq. (7)). However, it has been reported that the pencil beam dose calculation algorithm had limitations in accurately modeling scatter in the range compensator and patient (2–3% differences between the measurements and the estimations).^12^ Newhauser et al. has also reported that uncertainty of CPSF is approximately 1% for proton treatments of prostate cancer.^13^

By considering all the sources of uncertainty above, the estimated uncertainty by the correction‐based model for our system is about 2% for large field and 3.5% for small field (error propagation of all uncertainties; ROF = 0.5%, SOBPF = 0.5%, RSF = 1.0%, ISF_OCF_ = 0.5%, OCR = 1.0% for central area, FSF = 1.0% for large field or 3.0% for small field (estimated for 2 × 2 cm^2^ < field size < 5 × 5 cm^2^ from a publication using photon beams;^14^ a further study is required using proton beams), and GACF = 0.5%. The uncertainty of 2.0% for large field is equivalent to about 2 SDs (0.94% × 2) of our measurements. For the analytical models, these are about 2.5% for large field (also equivalent to 2 SDs of our measurements; 1.20% × 2) and 4.0% for small field (about 1.0% uncertainty of the basic model and source shift added to the uncertainty of ROF·SOBPF·RSF) as summarized in Table 4. In addition, uncertainty for CSF and PSF is considered clinically.

**Table 4:** Uncertainty analysis for the output prediction models for the compact proton system

Source of uncertainty	Correction‐based model	Analytical model
ROF	0.5%	1.5%^a^
SOBPF	0.5%	
RSF	1.0%	
ISF_OCF_	0.5%	0.5%
OCR	1.0%	1.0%
FSF		
Large field (>5 × 5 cm^2^)	1.0%	1.0%
Small field (≤5 × 5 cm^2^)	Up to 3.0%	Up to 3.0%
GACF	0.5%	0.5%
Total (error propagation)	2.0% for large field ~3.5% for small field	2.5% for large field ~4.0% for small field

a Combined uncertainty of the basic model (the first term of Eqs. (8) and (9)) and the source shift (the second term) which is equivalent to ROF × SOBPF × RSF.

## 5 Conclusion

For all options, all three models had acceptable predictions (<3% difference between prediction and measurement). The differences between model A, model B, and model C are statistically insignificant. In general, the model A has a potential to more accurately predict output if a larger dataset for commissioning is used. Field sizes less than 5 × 5 cm^2^ should have their output measured directly to ensure accuracy. System‐specific changes in output, such as the OCR and GACF for our Mevion system, should be investigated for each individual system. It is concluded that the models can be comparably used for the compact passively scattered proton therapy system.

## Conflict of Interest

The authors declare no conflict of interest.
